# Supplementary material for: Development and validation of a machine learning-based predictive model for clinical remission in Crohn’s disease patients receiving Adalimumab therapy
Source: PLoS One. 2025 Sep 19;20(9):e0331447. doi: 10.1371/journal.pone.0331447 (PMC12448358; doi:10.1371/journal.pone.0331447)
Supplement: Attachment — Submitted filename: Authors Response to editor.pdf [file pone.0331447.s003.pdf]

Details in revision according to editor's comments and suggestions on the manuscript PONE-D-25-14568

Title of manuscript: Development and Validation of a Machine Learning-Based Predictive Model for Clinical Remission in Crohn's Disease Patients Receiving Adalimumab Therapy

Dear Editor,

Thank you very much for organizing the review of the manuscript. We also thank both reviewers for their constructive comments to help improve the quality of the manuscript. The reviewer's comments were carefully considered in revision and modified/added texts are marked in red in the revised manuscript. Detailed responses to comments are summarized below:

Reviewer #1

Comment A1: Please ensure that your manuscript meets PLOS ONE's style requirements, including those for file naming. The PLOS ONE style templates can be found at https://journals.plos.org/plosone/s/file?id=wjVg/PLOSOne_formatting_sample_main_body.pdf andhttps://journals.plos.org/plosone/s/file?id=ba62/PLOSOne_formatting_sample_title_authors_affiliations.pdf

Reply A1: Thank you for your valuable suggestions. The article has been revised in accordance with the requirements of the PLOS ONE journal.

Comment A2: Please note that PLOS ONE has specific guidelines on code sharing for submissions in which author-generated code underpins the findings in the manuscript. In these cases, all author-generated code must be made available without restrictions upon publication of the work. Please review our guidelines at https://journals.plos.org/plosone/s/materials-and-software-sharing#loc-sharing-code and ensure that your code is shared in a way that follows best practice and facilitates reproducibility and reuse.

Reply A2: Thank you for your valuable feedback. When we uploaded the manuscript, we had already uploa ded all our codes, including the codes for 6 types of machine learning and SHAP analysis plots. They are i n the Python Jupyter file format and contain detailed code comments. Everyone can download and run them.

Comment A3: Thank you for stating in your Funding Statement:

[Funding This work was supported by the National Natural Science Foundation of China (Grant number 82470586), and the grantee is Dr. Feihong Deng.].

Please provide an amended statement that declares *all* the funding or sources of support (whether external or internal to your organization) received during this study, as detailed online in our guide for authors at

http://journals.plos.org/plosone/s/submit-now. Please also include the statement "There was no additional external funding received for this study." in your updated Funding Statement.

Please include your amended Funding Statement within your cover letter. We will change the online submission form on your behalf.

Reply A3: Thank you for your careful review and valuable suggestions regarding the Funding Statement. We have thoroughly revised the financial disclosure in accordance with the guidelines provided by PLOS ONE (http://journals.plos.org/plosone/s/submit-now), ensuring all required information is included.

The updated Financial Disclosure Statement now includes:

Specific grant number (82470586) and the full name of the funding agency (National Natural Science Foundation of China).

Initials of the author who received the award (F. Deng).

Confirmation of no commercial funders or authors receiving support from commercial entities.

The URL of the sponsor's website (http://www.nsfc.gov.cn/).

A clear statement that the funders had no role in study design, data collection and analysis, decision to publish, or manuscript preparation.

We appreciate your guidance in ensuring compliance with the journal's requirements. Please let us know if any further adjustments are needed.

Comment A4: When completing the data availability statement of the submission form, you indicated that y ou will make your data available on acceptance. We strongly recommend all authors decide on a data sharin g plan before acceptance, as the process can be lengthy and hold up publication timelines. Please note that, though access restrictions are acceptable now, your entire data will need to be made freely accessible if you r manuscript is accepted for publication. This policy applies to all data except where public deposition woul d breach compliance with the protocol approved by your research ethics board. If you are unable to adhere to our open data policy, please kindly revise your statement to explain your reasoning and we will seek the editor's input on an exemption. Please be assured that, once you have provided your new statement, the asse ssment of your exemption will not hold up the peer review process.

Reply A4: Thank you for your feedback. Our original data has been uploaded. Considering the privacy and ethical issues of the patients, the names have been removed and only the clinical and test indicators of the patients at 12 weeks and 48 weeks have been retained. We agree to make the data public before the article is accepted.

Comment A5: PLOS requires an ORCID iD for the corresponding author in Editorial Manager on papers su

bmitted after December 6th, 2016. Please ensure that you have an ORCID iD and that it is validated in Editorial Manager. To do this, go to ‘Update my Information’ (in the upper left-hand corner of the main menu), and click on the Fetch/Validate link next to the ORCID field. This will take you to the ORCID site and allow you to create a new iD or authenticate a pre-existing iD in Editorial Manager.

Reply A5: Thank you for bringing the ORCID iD requirement to our attention. We confirm that the corresponding author’s ORCID iD has been updated and validated in Editorial Manager as per PLOS guidelines.

Comment A6: Please include a copy of Table 1 which you refer to in your text on page 6.

Reply A6: Thank you for pointing out the missing Table 1. We have added the table to the manuscript as requested.

We tried our best to improve the manuscript and made some changes in the manuscript. These changes will not influence the content and framework of the paper. And here we not only listed the changes but also marked the revised portions in red in the revised paper. We appreciate for Editors/Reviewers' warm work earnestly, and hope that the correction will meet with approval. Once again, thank you very much for your comments and suggestions.

Yours sincerely,

Dr. Feihong Deng

19 Jun 2025
