# Supplementary material for: Development and validation of a machine learning-based predictive model for clinical remission in Crohn’s disease patients receiving Adalimumab therapy
Source: PLoS One. 2025 Sep 19;20(9):e0331447. doi: 10.1371/journal.pone.0331447 (PMC12448358; doi:10.1371/journal.pone.0331447)
Supplement: Attachment — Submitted filename: Authors Response to Reviewers Comments-Reviewer 1.pdf [file pone.0331447.s004.pdf]

Details in revision according to reviewer1's comments and suggestions on the manuscript PONE-D-25-14568R1

Title of manuscript: Development and Validation of a Machine Learning-Based Predictive Model for Clinical Remission in Crohn's Disease Patients Receiving Adalimumab Therapy

Dear Editor,

Thank you very much for organizing the review of the manuscript. We also thank both reviewers for their constructive comments to help improve the quality of the manuscript. The reviewer's comments were carefully considered in revision and modified/added texts are marked in red in the revised manuscript. Detailed responses to comments are summarized below:

Reviewer #1

Comment A1: The section "Research Significance and Prospects" at the very end appears to be a form of "Related Work" section. I believe it would be better to include it after the "Introduction" section and make it more comprehensive

Reply A1: Thanks to the reviewers' comments, we have moved the research review in the "Research Significance and Prospects" section to the "Introduction" section, enabling readers to more clearly understand the significance of this study. Once again, we are grateful for your valuable suggestions, which have made our article more rigorous.

Comment A2: In "Baseline Data Statistics" subsection, there is no rationale or citation for selecting statistical significance at less than 0.01. An explanation would enrich it.

Reply A2: Thanks to the comments from the reviewers. This is a common statistical principle. In most basi c research and clinical studies, P < 0.05 is the widely accepted standard for statistical significance. Its core logic is to control the "probability of obtaining a significant result due to random error" within 5%, which i s a conventional consensus in the field of research. We chose a more stringent threshold of P < 0.01 for st atistical significance, rather than the conventional P < 0.05. This is because the study involves comparisons between groups of multiple baseline variables (such as gender, smoking history, Montreal classification, 8 lab oratory indicators, etc.). Multiple tests increase the probability of "false positive results" (Type I error). By l owering the threshold to P < 0.01, the probability of "false significance" associations caused by random erro r can be reduced. This is also a common strategy and consensus in clinical research for controlling the risk of multiple comparisons. Therefore, we did not provide an explanation.

Comment A3: The dataset is now available upon request. But Plos One requires the dataset to be fully public.

Reply A3: Thank you for the comments from the reviewers. This was indeed our mistake. When we submitted the manuscript, we uploaded the data and code as supporting information files. I'm not sure if the reviewers could access these data, but we have already uploaded them and stated in the manuscript that "All relevant data are within the manuscript and its supporting information files." We have also changed the status of the Data availability statement in the manuscript to "All relevant data are within the manuscript and its supporting information files." If you have any questions, please feel free to contact us at any time.

We tried our best to improve the manuscript and made some changes in the manuscript. These changes will not influence the content and framework of the paper. And here we not only listed the changes but also marked the revised portions in red in the revised paper. We appreciate for Editors/Reviewers' warm work earnestly, and hope that the correction will meet with approval. Once again, thank you very much for your comments and suggestions.

Yours sincerely,

Dr. Feihong Deng

9 Aug 2025
